# Perspectives on the 2014 ESC/EACTS Guidelines on Myocardial Revascularization: Fifty Years of Revascularization: Where Are We and Where Are We Heading?

**DOI:** 10.1007/s12265-015-9632-6

**Published:** 2015-05-19

**Authors:** Francesco Costa, Sara Ariotti, Marco Valgimigli, Philippe Kolh, Stephan Windecker, on behalf of the Task Force on Myocardial Revascularization of the European Society of Cardiology (ESC) and the European Association for Cardio-Thoracic Surgery (EACTS)

**Affiliations:** Thoraxcenter, Erasmus Medical Center, 3015 CE Rotterdam, The Netherlands; Department of Clinical and Experimental Medicine, Policlinico “G. Martino”, University of Messina, Messina, Italy; Division of Cardiology of the Department of Medicine, University of Verona, Verona, Italy; Cardiovascular Surgery Department, University Hospital (CHU, ULg) of Liege, Liege, Belgium; Department of Cardiology, Bern University Hospital, Bern, Switzerland

**Keywords:** PCI, CABG, Guidelines, Coronary stent, DES, BMS

## Abstract

The joint European Society of Cardiology and European Association of Cardio-Thoracic Surgery (ESC/EACTS) guidelines on myocardial revascularization collect and summarize the evidence regarding decision-making, diagnostics, and therapeutics in various clinical scenarios of coronary artery disease, including elective, urgent, and emergency settings. The 2014 document updates and extends the effort started in 2010, year of the first edition of these guidelines. Importantly, this latest edition provides a systematic review of all randomized clinical trials performed since 1980, comparing different strategies of myocardial revascularization, including coronary artery bypass graft (CABG), balloon angioplasty, percutaneous coronary intervention (PCI) with bare-metal stents (BMS) and first- and second-generation drug-eluting stents (DES). This review aims to highlight the most relevant novelties introduced by the 2014 edition of the ESC/EACTS myocardial revascularization guidelines as compared with the previous edition and to describe similarities and differences with the American societies’ guidelines.

**Electronic supplementary material:**

The online version of this article (doi:10.1007/s12265-015-9632-6) contains supplementary material, which is available to authorized users.

## Introduction

The most recent edition of the European Society of Cardiology (ESC) and the European Association for Cardio-Thoracic Surgery (EACTS) joint guidelines on myocardial revascularization celebrates the 50th anniversary of the first coronary artery bypass graft (CABG) procedure [1, 2]. The first percutaneous coronary revascularization procedure was performed only 13 years thereafter, in 1977. Since their first introduction, revascularization techniques gained expertise and clinical relevance worldwide, becoming one of the most commonly performed interventions in modern medicine. The ESC joint guidelines inform European and non-European practitioners since the early 2000s and represent the endeavor of dozens of clinical and research professionals in the field of cardiovascular medicine. The 2014 edition of the ESC/EACTS revascularization guidelines provides a concise and updated summary of the evidence surrounding the value of revascularization in various clinical scenarios, including elective, urgent, and emergency settings. Unique to this edition, they provide a systematic review of all randomized clinical trials performed since 1980, comparing different strategies of myocardial revascularization, including CABG, balloon angioplasty, percutaneous coronary intervention (PCI) with bare-metal stents (BMS) and first- and second-generation drug-eluting stents (DES).

The following perspective paper is intended to highlight the most relevant novelties in the field of revascularization introduced in these guidelines, as compared with the previous 2010 edition [3]. In addition, similarities and differences with respect to the American societies’ guidelines on myocardial revascularization are discussed whenever proper [4–9].

## The Heart Team: from Inception to Mainstream

The 2010 edition of the ESC guidelines introduced and strongly empowered the concept of the Heart Team. This has been a great achievement whereby all relevant cardiac specialties and heart care providers are brought together to choose the best revascularization modality for each single patient. Current guidelines further extend the importance of the Heart Team discussion, by inciting the development of shared institutional protocols, in order to better select the patients that deserve a multidisciplinary evaluation, saving time, resources, and delays of urgent procedures, especially in centers without on-site surgery. American guidelines also advocate the institution of the Heart Team, indicating the need for multidisciplinary discussion in patients with left main coronary artery disease (CAD) or complex multivessel CAD.

## Applying Risk Scores in Practice

Aiming at achieving the best revascularization modality for each individual patient, the 2014 ESC/EACTS revascularization guidelines have updated and expanded the risk score section. The Society of Thoracic Surgeons (STS) score is recognized as the appropriate, recommended tool to stratify surgical risk during CABG, whereas the role of the EuroScore has been reconsidered and its use is no longer indicated, based on the concern that it overestimates the surgical risk (Table 1). However, the newly introduced EuroScore II overcomes this limitation, and its use should be preferred over the first iteration of this surgical risk score.

**Table 1:** Comparison among guidelines indications for risk scoring

	ESC GL 2014		ESC GL 2010		American societies’ GL
	CABG	PCI	CABG	PCI	CABG and PCI
STS score	I B	–	I B	–	• IIa B^a^
EuroScore	III B	III C	I B	IIb B	–
EuroScore II	IIa B	IIb C	–	–	–
SYNTAX	I B	I B	III B	IIa B	• IIa B^a^
SYNTAX II	IIa B	IIa B	–	–	–

*ESC* European Society of Cardiology, *GL* guidelines

^a^From the 2011 ACCF/AHA/SCAI PCI Guideline [4]: this document specifies that calculation of STS and SYNTAX is reasonable in patients with unprotected left main and complex CAD

The Synergy Between Percutaneous Coronary Intervention with TAXUS and Cardiac Surgery (SYNTAX) score, introduced in the previous edition, is now recommended for the risk stratification of patients who undergo revascularization (CABG vs. PCI). The more recent SYNTAX II score has been introduced in this guidelines edition for the very first time (Table 1). The latter is a combination of anatomical and clinical factors that were found to be superior to the conventional SYNTAX score in guiding decision-making between CABG and PCI [10].

Among the aforementioned scores, STS and SYNTAX are also mentioned in the American guidelines as reasonable tools to guide the decision-making of the revascularization modality.

## Revascularization of the Left Main Coronary Artery

There is increasing evidence that both CABG and PCI may provide effective treatment for selected patients with left main CAD, especially those with an overall low to intermediate anatomical complexity. A prespecified analysis of the SYNTAX trial evaluated a subgroup of patients with predominant distal left main disease [11]. Despite its limited statistical power, this study showed that CABG and PCI had a comparable rate of the primary endpoint—a composite of death, myocardial infarction, stroke, and repeat revascularization—in the low and intermediate SYNTAX tertile (SYNTAX score ≤22 and SYNTAX score 23–32). In contrast, it observed a numerical increase of deaths and a significant increase of repeat revascularizations in the PCI group with the highest SYNTAX tertile (SYNTAX >32). In keeping with this, the PRECOMBAT trial showed comparable outcomes at 1 and 2 years in patients with LM disease treated with CABG or PCI [12].

Based on these data, the indication for PCI of left main CAD with low anatomical complexity (SYNTAX score ≤22) has been upgraded and now equated to CABG, whereas in anatomies with intermediate (SYNTAX score 23–32) complexity, PCI should be considered, but CABG remains the preferred revascularization modality (Table 2).

**Table 2:** Recommendation for the type of revascularization (CABG or PCI) in patients with SCAD and left main coronary artery disease with suitable anatomy and low predicted surgical mortality

	ESC GL 2014		ESC GL 2010		American societies’ GL^c^
	CABG	PCI	CABG	PCI	PCI
SYNTAX score ≤22	I B	I B	I A	IIa/b B^a^	• IIa B—if low risk of PCI complications and significantly increased surgical risk (e.g., STS ≥5 %)
SYNTAX score 23–32	I B	IIa B	I A	IIb B^b^	• IIb B—if low to intermediate risk of PCI complications and increased surgical risk (e.g., STS >2 %)
SYNTAX score >32	I B	III B	I A	III B	• III B—if unfavorable anatomy for PCI and good candidates for CABG

*GL* guidelines

^a^Indication IIa B for left main lesion at ostium/shaft. Indication IIb B for left main lesion at distal bifurcation

^b^Indication for left main disease associated to two- or three-vessel disease and a SYNTAX score ≤32

^c^Indication to improve survival with revascularization as compared to medical therapy

Similar to the 2010 edition, the most recent revascularization guidelines reiterate the contraindication to the elective treatment of left main CAD with PCI, in case of high anatomical complexity (SYNTAX >32) in patients who have an acceptable surgical risk (Table 2). Properly powered trials evaluating the outcomes of the new-generation DES vs. CABG are still lacking. The EXCEL trial is expected to provide important insights on this matter.

At variance from the European document, the American societies’ guidelines recommend CABG for the treatment of left main CAD and suggest PCI as an alternative in patients with an increased surgical risk and an amenable anatomy [4, 6, 9] (Table 2).

## Revascularization of the Proximal Left Anterior Descending Artery

PCI indication was upgraded for the treatment of the proximal left anterior descending artery (LAD) disease (Table 3). In this regard, one study comparing PCI with DES and CABG in patients with isolated proximal LAD disease demonstrated similar outcomes over a 10-year follow-up [13]. Similarly, no survival benefit with CABG vs. PCI was observed for the treatment of two-vessel disease including proximal LAD. Accordingly, PCI is now equally recommended as CABG for the treatment of proximal LAD alone as well as in the context of a two-vessel disease. This recommendation slightly diverges from the American document, which considers CABG superior for the treatment of two-vessel disease including the proximal LAD [6, 9].

**Table 3:** Recommendation for the type of revascularization (CABG or PCI) in patients with SCAD and proximal left anterior descending coronary artery disease with suitable anatomy and low predicted surgical mortality

	ESC GL 2014		ESC GL 2010		American societies’ GL^a^
	CABG	PCI	CABG	PCI	
One-vessel disease	I A	I A	I A	IIa B	• IIa B for CABG with LIMA • IIb B for PCI
Two-vessel disease	I B	I C	I A	IIa B	• I B for CABG • IIb B for PCI

*GL* guidelines

^a^Indication to improve survival with revascularization as compared to medical therapy

## Revascularization for Three-Vessel Coronary Artery Disease

At variance with previous guidelines, PCI is now equally recommended as CABG for the treatment of three-vessel disease with a low anatomical complexity (SYNTAX score ≤ 22) [14–16], whereas in more complex anatomies (SYNTAX score >22), PCI is still contraindicated (Table 4).

**Table 4:** Recommendation for the type of revascularization (CABG or PCI) in patients with SCAD and three-vessel coronary artery disease with suitable anatomy and low predicted surgical mortality

	ESC GL 2014		ESC GL 2010		American societies’ GL
	CABG	PCI	CABG	PCI	
SYNTAX score ≤22	I A	I B	I A	IIa B	• IIa B—it is reasonable to choose CABG over PCI in patients with complex three-vessel disease (e.g., SYNTAX >22) who are good candidates for CABG
SYNTAX score 23–32	I A	III B	I A	III A	
SYNTAX score >32	I A	III B	I A	III A	

These recommendations are largely based on the results of the 5-year follow-up of the SYNTAX trial. CABG showed better outcomes in the overall three-vessel disease population, whereas PCI demonstrated to be a reasonable alternative in those with a low SYNTAX score ≤22, although at the price of an increased risk of repeat revascularization [16]. The risk of stroke in this population has been shown to be lower after PCI as compared to CABG. The SYNTAX trial tested the effect of TAXUS stent implantation, a first-generation DES. Given the overwhelming evidence showing superior outcomes when newer generation DES are compared to paclitaxel-eluting stent in patients undergoing coronary stent implantation, it remains likely that the use of newer generation DES may further improve the efficacy and safety of PCI when compared to CABG in this high-risk population. This hypothesis requires validation in prospective clinical trials.

## Revascularization in Patients with Comorbidities

The 2014 edition largely focuses on revascularization modalities in patients with various comorbidities, especially diabetes mellitus and chronic kidney disease.

CABG is strongly recommended over PCI for patients with diabetes and multivessel disease, provided surgical risk is acceptable. In cases where a percutaneous treatment is indicated, new-generation DES should be preferred over bare-metal stents [15, 17]. In keeping with this, American guidelines also indicate CABG as the treatment of choice in patients with diabetes and multivessel disease [9].

These recommendations are mainly based on the results of the FREEDOM trial [15]; this study randomized diabetic patients with multivessel disease to CABG or PCI + DES and found a significantly higher rate of the primary endpoint—a composite of death, myocardial infarction, and stroke—in the PCI group. Moreover, death and myocardial infarction occurred more frequently in the PCI group, whereas stroke rate was higher after CABG. Similar results are provided by a recent meta-analysis that confirmed a survival benefit of CABG over PCI in diabetic patients with multivessel disease, irrespectively the use of DES or BMS [17].

As in diabetic patients, new guidelines recommend new-generation DES over BMS in patients with chronic kidney disease (CKD). In patients with CKD and multivessel disease, CABG is still the treatment of choice, with off-pump CABG that may be preferred over the on-pump approach [18].

The lack of properly powered randomized trials comparing different revascularization modalities is notable in this setting. In patients at risk of contrast-induced acute kidney injury, the use of short-term, high-dose statin therapy should be considered [19].

## Antiplatelet Therapy and Revascularization

New guidelines no longer indicate to pretreat with clopidogrel all patients scheduled for a diagnostic coronary angiogram (Supplementary Table 1); indeed, pretreatment did not outperform no-pretreatment option in a meta-analysis of 37,814 patients, which included both prospective controlled studies and retrospective registry data [20]. Differently, it remains reasonable to pretreat patients with known coronary anatomy scheduled for PCI. Pretreatment may still be considered in cases where the probability of CAD is high and the anticipated need for urgent CABG unlikely.

The indications for dual antiplatelet therapy (DAPT) duration have been updated (Table 5). In patients with spontaneous coronary artery dissection (SCAD) receiving a DES, 6-month DAPT is now recommended. A shortened DAPT duration may be considered in case of high bleeding risk. This indication was extrapolated from several trials comparing standard or prolonged DAPT regimens with shorter courses, which eventually failed to demonstrate a benefit from a prolonged DAPT, but rather observed an increased risk of bleeding after a longer therapy [21, 22].

**Table 5:** Indication to antiplatelet therapy after stenting in European and American guidelines

ESC GL 2014	ESC GL 2010	American societies’ GL
No-ACS patient • New DES → 6 months • BMS → at least 1 month ACS patient • New DES → up to 12 months • BMS → up to 12 months	No-ACS patient • DES → 6 to 12 months • BMS → at least 1 month ACS patient • DES → 12 months • BMS → 12 months	No-ACS patient • DES → at least 12 months • BMS → at least 1 month ACS patient • DES → at least 12 months • BMS → at least 12 months
Special considerations - Shorter DAPT (<6 months) may be considered in patients with high bleeding risk. - DAPT may be used for more than 6 months in patients at high ischemic risk and low bleeding risk. - In patients with SCAD and atrial fibrillation with indication to anticoagulation and low bleeding risk, triple therapy should be considered for at least 1 month, irrespective of the stent used, followed by dual therapy with (N)OAC + ASA or clopidogrel up to 12 months. In patients with ACS and atrial fibrillation with indication to anticoagulation and low bleeding risk, triple therapy should be considered for at 6 months, irrespective of the stent used, followed by dual therapy with (N)OAC + ASA or clopidogrel up to 12 months. In case of high bleeding risk, triple therapy should be considered for 1 month, irrespective the clinical presentation and the type of stent used, followed by dual therapy with (N)OAC + ASA or clopidogrel.	Special considerations - In patients with a compelling indication for long-term anticoagulation, BMS implantation or stand-alone balloon angioplasty or CABG should be preferred over DES to restrict the duration of triple therapy to 1 month. - Triple therapy should be prescribed for the shortest necessary duration.	Special considerations - In patients receiving BMS for a non-ACS indication, at increased risk of bleeding; clopidogrel (should be given for a minimum of 2 weeks). - If the risk of morbidity from bleeding outweighs the anticipated benefit afforded by a recommended duration of P2Y_12_ inhibitor therapy after stent implantation, earlier discontinuation (e.g., <12 months) of P2Y_12_ inhibitor therapy is reasonable. - Continuation of clopidogrel, prasugrel, or ticagrelor beyond 12 months may be considered in patients undergoing placement of DES.

If the individual ischemic risk is high and bleeding risk is low, DAPT may be prolonged beyond 6 months. American guidelines (GL) recommend at least 12 months of therapy in patients with SCAD treated with DES, unless at high bleeding risk (Supplementary Table 1).

The novel P2Y_12_ inhibitors, prasugrel or ticagrelor, are recommended as first-line treatment during acute coronary syndrome (ACS), whereas clopidogrel should be used only when prasugrel and ticagrelor are not available (Supplementary Table 2 and 3). American guidelines are less prescriptive and state that it is reasonable to prefer ticagrelor over clopidogrel, provided ischemic risk is high and an early invasive strategy is planned, whereas they state that prasugrel should be preferred over clopidogrel if the bleeding risk is low [8].

Importantly, after the presentation of the ACCOAST trial, the European GL now contraindicate the pretreatment with prasugrel in patients with non-ST-segment elevation-ACS (NSTE-ACS) and unknown coronary anatomy, given the increased risk of major bleeding and the lack of ischemic benefit [23]. Notably, the administration of P2Y_12_ inhibitors before catheterization in ST segment elevation myocardial infarction (STEMI) is recommended, and ideally, they should be administered at the time of the first medical contact. This recommendation is in keeping with American guidelines and is supported by a small randomized study [24], two observational studies [25, 26], and one meta-analysis [20] showing a reduction of death and MACE without increase of bleeding, in STEMI patients pretreated with clopidogrel.

## Anticoagulant Therapy and Revascularization

The anticoagulation section has also been revised with some novelties regarding the management of bivalirudin and use of novel oral anticoagulants (NOAC).

In the previous edition of the European guidelines as well as in Americans’ [7], bivalirudin had a first-class indication as recommended anticoagulant during PCI in STEMI compared to heparin plus glycoprotein IIb/IIIa inhibitors (GPI) (Supplementary Table 3). However, the recently published HEAT PPCI trial [27] did not show a reduction of bleeding in patients treated with bivalirudin as compared to heparin alone. Accordingly, the current document gives bivalirudin a second-class indication as anticoagulant in the setting of STEMI as compared to heparin without GPI. While this new indication has been largely interpreted as downgrading, it should be emphasized that previous guidelines set a recommendation of bivalirudin instead of unfractionated heparin (UFH) plus routine use of glycoprotein IIb/IIIa inhibitors, whereas the more recent availability of comparative effectiveness data of bivalirudin versus UFH alone has made possible to provide new recommendations of bivalirudin as contrasted to UFH without routine use of glycoprotein IIb/IIIa inhibitors.

In the NSTE-ACS setting, bivalirudin administered during the PCI and prolonged for up to 4 h thereafter has a class IA indication as an alternative to UFH + GPI and is recommended whenever available (Supplementary Table 2). This indication is mainly driven by the results of the ACUITY and ISAR-REACT 4 trials where bivalirudin compared to UFH + GPI showed a similar efficacy and a better bleeding profile [28, 29]. It has to be highlighted that most of the evidence in this setting comes from trials testing bivalirudin versus UFH + GPI, a combination that is no longer routinely applied; thus, confirmation of bivalirudin benefit in properly powered trials is still needed [30].

In elective patients instead, bivalirudin is recommended in case of heparin-induced thrombocytopenia (Supplementary Table 1).

In addition, a prolonged infusion of bivalirudin should now be considered for up to 4 h after PCI, based on the concern of an increased risk of acute stent thrombosis.

With respect to NOACS, these guidelines also mention the possibility of adding a third agent, namely, rivaroxaban, on top of the standard DAPT with aspirin and clopidogrel for ACS patients treated with PCI in patients at low bleeding risk. This is based on the recent ATLAS-ACS2 trial that observed a mortality benefit from a triple therapy consisting of ASA, clopidogrel, and low-dose rivaroxaban (i.e., 2.5 twice daily) in patients recently treated for ACS [31]. However, this was at an expense of an increase of severe bleeding, and no data currently exists on the value of rivaroxaban when tested in patients taking the new P2Y_12_ inhibitors.

The lack of formal guidance with respect to DAPT duration in patients requiring long-term oral anticoagulation has now been overcome with this edition of the guidelines. Also in this setting, new-generation DES should be preferred over BMS, provided that the bleeding risk is low (HAS BLED ≤2).

In patients with SCAD with absolute indication to anticoagulation and low bleeding risk (HAS BLED ≤2), the duration of the triple therapy—consisting of aspirin, clopidogrel, and a (N)OAC—should be of at least 1 month and ideally continued up to 12 months, whereas in patients presenting ACS, triple therapy should be considered for 6 to 12 months, irrespective of the stent used. Importantly, for patients at high bleeding risk (HAS BLED > 2), the duration of triple therapy should be of 1 month irrespective the presentation (i.e., SCAD or ACS) and the type of stent used.

## Recommendations on New-Generation Drug-Eluting Stents

At variance with the previous document, which listed several relative limitations to the use of DES, in the current edition, second-generation drug-eluting stents receive an unrestricted indication of use (Table 6). To support this, a network meta-analysis recently published by Windecker et al. included more than 100 studies comparing revascularization and medical therapy in patients with stable coronary artery disease [32]. This meta-analysis showed a survival benefit for CABG as compared to medical treatment, in keeping with previous data. In addition, new-generation DES, but not balloon angioplasty, BMS, or first-generation DES, showed a survival improvement compared to medical therapy. This is the first report that demonstrates a reduction of mortality in SCAD with percutaneous revascularization. A possible biological explanation for the survival benefit of these new stents could be related to the lower risk of myocardial infarction and stent thrombosis. This is consistent with other recent studies that showed a dramatic improvement in cardiac outcomes, including cardiac survival, myocardial infarction, and stent thrombosis with cobalt-chromium everolimus-eluting stents (new-generation devices), compared with both first-generation DES and bare-metal stents [33, 32, 34].

**Table 6:** Position of European and American guidelines with respect to the use of drug-eluting stents

ESC GL 2014	ESC GL 2010	American societies’ GL
• Unrestricted use of new-generation DES	The use of DES is relatively contraindicated if • Clinical history difficult to obtain, especially in the setting of acute severe clinical conditions (STEMI or cardiogenic shock). • Expected poor compliance with DAPT, including patients with multiple comorbidities and polypharmacy. • Non-elective surgery required in the short-term that would require interruption of DAPT. • Increased risk of bleeding. • Known allergy to ASA or clopidogrel/prasugrel/ticagrelor. • Absolute indication for long-term anticoagulation.	• Before implantation of DES, the interventional cardiologist should discuss with the patient the need for and duration of DAPT and the ability of the patient to comply with and tolerate DAPT. • Balloon angioplasty or BMS should be used in patients with high bleeding risk, inability to comply with 12 months of DAPT, or anticipated invasive or surgical procedures within the next 12 months, during which time DAPT may be interrupted. • DES should not be implanted if the patient is not likely to be able to tolerate and comply with prolonged DAPT or this cannot be determined before stent implantation. • DES should not be implanted if the patient is not likely to be able to tolerate and comply with prolonged DAPT or this cannot be determined before stent implantation.

According to this evidence, new guidelines recommend new-generation DES as default in all clinical conditions and lesion subsets. In addition, the previous concerns associated with early DAPT cessation are not confirmed by recent data, and new-generation DES are recommended over BMS also in patients who may require earlier discontinuation of antiplatelet therapy. American guidelines profoundly diverge from the current ESC position and list several, strong contraindication to DES use as the inability, or the unproven ability, to comply or tolerate a prolonged DAPT (Table 6). It is worth mentioning that American guidelines on percutaneous coronary intervention date back to 2011, so it is possible that these differences will be in part leveled with updated editions.

## Conclusions and Future Perspectives

The 2014 edition of the ESC/EACTS guidelines implements important novelties including the unrestricted indication to new-generation DES, the modulation of DAPT duration according to clinical presentation, and the new indications for the treatment of left main and three-vessel CAD. The value of bioresorbable polymer or no-polymer DES over more conventional durable polymer DES remains under evaluation, and whether this more sophisticated technology will translate into improved patient outcomes remains unsettled. The use of bioresorbable vascular scaffolds, instead of permanent metallic DES, while highly promising for restoring physiological vessel motion long-term after intervention remains also a matter for ongoing research. The recent DAPT and PEGASUS trials explored the effectiveness of a long-term treatment with a P2Y_12_ inhibitor, clopidogrel/prasugrel for the first, ticagrelor for the latter, showing ischemic benefit for reductions of patient and device-oriented non-fatal endpoints, counterbalanced by higher bleeding rates [35, 36]. The optimal DAPT type and duration, which maximize the benefits in terms of ischemic protection and minimize the risks in terms of bleeding, will be most likely based on the individual patient’s risk profile. It is probable that in the near future, strategies based on weighting patients risk by the use of clinical (i.e., risk scores), biochemical (i.e., circulating biomarkers), or genetic-based tools (i.e., gene polymorphisms) will help physicians to better individualize this treatment.

The MATRIX program is the first large multicenter study showing the superiority of the radial as compared to femoral access, for the reduction of a net clinical benefit endpoint, driven by lower major bleeding and mortality rates [30, 37–41]. Future recommendations will most likely further upgrade the use of radial over femoral route for ACS patients undergoing invasive management, which will have implications in terms of training programs as well as health care quality assessment.

The decision to revascularize a given lesion or vessel in the near future will likely depend even more on functional parameters. Some techniques have already demonstrated solid results (i.e., fractional flow reserve—FFR) whereas some more recent potentially simplified iterations look promising (i.e., instantaneous wave-free ratio—IFR). The results of future studies evaluating the incremental value of a routine functional evaluation and imaging-based stent optimization algorithm may further optimize outcomes and patient selection in revascularization procedures. The recent COSIRA study reported the efficacy of a coronary sinus reducer to relieve symptoms in patients with refractory angina not amenable for revascularization. This device may serve the growing proportion of patients that remains symptomatic despite maximal antianginal therapy [42]. However, even if the concept of a mechanical treatment of refractory angina is intriguing, more informative clinical studies are needed to confirm the role of such device in clinical practice.

## Electronic Supplementary Material

ESM 1 (DOCX 19 kb)

ESM 2 (DOCX 23 kb)

ESM 3 (DOCX 22 kb)
