# Supplementary material for: Perspectives on the 2014 ESC/EACTS Guidelines on Myocardial Revascularization: Fifty Years of Revascularization: Where Are We and Where Are We Heading?
Source: J Cardiovasc Transl Res. 2015 May 19;8(4):211–20. doi: 10.1007/s12265-015-9632-6 (PMC4473080; doi:10.1007/s12265-015-9632-6)
Supplement: Supplementary file 1 — (DOCX 19 kb) [file 12265_2015_9632_MOESM1_ESM.docx]

Supplementary Table 1: Recommendations for antithrombotic treatment in stable coronary artery disease patients undergoing PCI:

|  | **ESC GL 2014** | | **ESC GL 2010** | | **American Societies GL** | |
| --- | --- | --- | --- | --- | --- | --- |
| Antiplatelet Therapy | ASA | **I B** | ASA | **I B** | ASA | I B |
|  | Clopidogrel | **I A** | Clopidogrel | **I A** | Clopidogrel | **I B** |
|  | Clopidogrel pretreatment (600 mg) for elective PCI once coronary anatomy is known | **I A** | Clopidogrel pretreatment  (300 or 600 mg) | **I C** | Clopidogrel pretreatment  (600 mg) | **I B** |
|  | GP IIb/IIIa  (bailout only) | **IIa C** | GP IIb/IIIa  (bailout only) | **IIa C** | In patients undergoing elective PCI treated with UFH and not pretreated with clopidogrel it is reasonable to administer a GP IIb/IIIa inhibitor | **IIa B** |
|  |  |  |  |  | In patients undergoing elective PCI treated with UFH and pretreated with clopidogrel it might be reasonable to administer a GP IIb/IIIa inhibitor | **IIb B** |
|  | DAPT at least 1 month after BMS | **I A** | DAPT at least 1 month after BMS | **-** | DAPT at least 1 month after BMS (if patients at high bleeding risk at least 2 weeks) | **I B** |
|  | DAPT for 6 months after DES | **I B** | DAPT 6 to 12 months after DES | **-** | DAPT for at least 12 months after DES if patients are not at high bleeding risk | **I B** |
|  | DAPT < 6 months may be considered after DES if High bleeding risk | **IIb A** | n.a. | **-** | If the risk of morbidity from bleeding outweighs the anticipated benefit afforded by a recommended duration of P2Y_12_ inhibitor therapy after stenting , earlier discontinuation  (<12 months) is reasonable | **II a C** |
|  | Life-Long single antiplatelet therapy | **I A** | n.a. | **-** | Life-Long single antiplatelet therapy | **I A** |
|  | DAPT may be used for more than 6 months in patients at high ischemic risk and low bleeding risk | **IIb C** | n.a. | **-** | Continuation of DAPT beyond 12 months may be considered in patients undergoing DES implantation | **IIb C** |
| Anticoagulant Therapy | UFH 70-100 U/Kg (if no GPI) | **I B** | UFH 70-100 U/Kg (if no GPI) | **I C** | UFH 70-100 U/Kg (if no GPI) | **I C** |
|  | Bivalirudin (0.75 mg/kg bolus + 1.75 mg/kg/h up to 4 hours after PCI) in case of Heparin Induced Thrombocytopenia | **I C** | If case of heparin-induced thrombocytopenia, neither UFH nor LMWH should be used because of cross- reactivity. In this case, bivalirudin is the best option. | **-** | For patients with heparin-induced thrombocytopenia, it is recommended that bivalirudin or argatroban be used to replace UFH. | **I B** |
|  | Bivalirudin (0.75 mg/kg bolus + 1.75 during PCI) in patients at high bleeding risk | **IIa A** | n.a. | **-** | For patients undergoing PCI, bivalirudin is useful as an anticoagulant with or without prior treatment with UFH | **I B** |
|  | Enoxaparin i.v. 0.5 mg/kg | **IIa B** | Enoxaparin i.v. 0.5 mg/kg | **IIa B** | Enoxaparin i.v. 0.5 to 0.75 mg/kg | **IIb B** |

GL: Guidelines

This table is based on the original table present in the ESC revascularization guidelines 2014. The data regarding the 2010 guidelines and American guidelines are adapted to allow comparison. The fields in which the comparison was not possible or could risk to distort the sense of the indications have been specified as “not applicable” (n.a.)
