# Supplementary material for: Perspectives on the 2014 ESC/EACTS Guidelines on Myocardial Revascularization: Fifty Years of Revascularization: Where Are We and Where Are We Heading?
Source: J Cardiovasc Transl Res. 2015 May 19;8(4):211–20. doi: 10.1007/s12265-015-9632-6 (PMC4473080; doi:10.1007/s12265-015-9632-6)
Supplement: Supplementary file 2 — (DOCX 23 kb) [file 12265_2015_9632_MOESM2_ESM.docx]

Supplementary Table 2: Recommendations for antithrombotic treatment in Non ST-segment Elevated Acute Coronary Syndrome patients undergoing PCI:

|  | **ESC GL 2014** | | **ESC GL 2010** | | **American Societies GL** | |
| --- | --- | --- | --- | --- | --- | --- |
| Antiplatelet Therapy | ASA is recommended for all patients without contraindications and continued long-term regardless of treatment strategy. | **I A** | ASA | **I C** | ASA in patients already taking daily aspirin and in patients not on aspirin | I B |
|  |  |  |  |  | ASA after PCI should be continued indefinitely | I A |
|  | P2Y_12_ Inhibitor is recommended in addition to ASA and maintained over 12 months unless contraindicated (eg, excessive bleeding risk), options are: | **I A** | n.a. | **-** | A loading dose of P2Y_12_ Inhibitor should be given to patients undergoing PCI with stenting, options include: | **I A** |
|  | Prasugrel in patients with known coronary anatomy proceeding to PCI, if no contraindications. | **I B** | Prasugrel | **IIa B** | Prasugrel | **I B** |
|  | Ticagrelor for patients at moderate-high risk of ischemic events regardless initial treatment strategy, if no contraindications. | **I B** | Ticagrelor | **I B** | Ticagrelor | **I B** |
|  | Clopidogrel only when prasugrel or ticagrelor are not available or contraindicated. | **I B** | Clopidogrel (600 mg loading dose as soon as possible) | **I C** | Clopidogrel | **I B** |
|  |  |  | Clopidogrel for 9-12 months after PCI | **I B** |  |  |
|  | GP IIb/IIIa antagonist should be considered for bailout situations or thrombotic complications. | **IIa C** | GP IIb/IIIa antagonists (in patients with evidence of high intracoronary thrombus burden: |  | GP IIb/IIIa inhibitor is useful at the time of PCI in patients with high-risk features (eg, elevated troponin) not treated with bivalirudin and not adequately pre-treated with clopidogrel. | **I A** |
|  |  |  | Abciximab | **I B** | GP IIb/IIIa inhibitor is reasonable at the time of PCI in patients with high-risk features (eg, elevated troponin) | **IIa B** |
|  |  |  | Tirofiban or Eptifibatide | **IIa B** | treated with UFH and adequately pre-treated with clopidogrel. |  |
|  | Pre-treatment with Prasugrel in patients with not known coronary anatomy is contraindicated | **III B** | n.a. | **-** | n.a. | **-** |
|  | Pre-treatment with GPIIb/IIIa antagonists is not recommended | **III A** | Upstream GP IIb/IIIa antagonists | **III B** | n.a. | **-** |
| Anticoagulant Therapy | Anticoagulant therapy is recommended for all patients in addition to antiplatelet therapy during PCI | **I A** | n.a. | **-** | An anticoagulant should be administered to patients undergoing PCI | **I C** |
|  | Anticoagulation is selected according to both ischemic and bleeding risks, and according to the safety-efficacy profile of the chosen agent | **I C** | n.a. | **-** | n.a. | **-** |
|  | Bivalirudin (0.75 mg/kg bolus + 1.75 mg/kg/h up to 4 hours after PCI) is recommended as alternative to UFH + GPIIb/IIIa receptor inhibitor during PCI | **I A** | Bivalirudin (monotherapy) in very high-risk of ischemia | **I B** | Bivalirudin is useful as an anticoagulant, for patients undergoing PCI with or without prior treatment with UFH | **I B** |
|  |  |  | Bivalirudin in medium- to high-risk of ischemia | **I B** |  |  |
|  | UFH is recommended as anticoagulant for PCI if patients cannot receive bivalirudin. | **I C** | UFH (+ GPIIb/IIIa antagonist) in very high risk of ischemia | **I C** | UFH is useful in patients undergoing PCI | **I C** |
|  |  |  | UFH in medium- to high-risk of ischemia | **I C** |  |  |
|  | In patients on fondaparinux a single bolus of UFH is indicated during PCI | **I B** | n.a. | **-** | Fondaparinux should not be used as the sole anticoagulant to support PCI. An additional anticoagulant with anti-IIa activity should be administered because of the risk of catheter thrombosis | **III C** |
|  | Enoxaparin should be considered as anticoagulant for PCI if patient pre-treated with subcutaneous enoxaparin. | **IIa B** | Enoxaparin in medium- to high-risk of ischemia | **IIa B** | Enoxaparin may be reasonable at the time of PCI in patients either treated with “upstream” subcutaneous enoxaparin or who have not received prior antithrombin therapy | **IIb B** |
|  |  |  | Enoxaparin in low-risk of ischemia | **IIa B** |  |  |
|  | Discontinuation of anticoagulation after procedure should be considered unless otherwise indicated | **IIa C** | n.a. | **-** | n.a. | **-** |
|  | Crossover of UFH and LMWH is not recommended | **III B** | n.a. | **-** | UFH should not be given to patients already receiving therapeutic subcutaneous enoxaparin | **III B** |

GL: Guidelines

This table is based on the original table present in the ESC revascularization guidelines 2014. The data regarding the 2010 guidelines and American guidelines are adapted to allow comparison. The fields in which the comparison was not possible or could risk to distort the sense of the indications have been specified as “not applicable” (n.a.)
