# Supplementary material for: Perspectives on the 2014 ESC/EACTS Guidelines on Myocardial Revascularization: Fifty Years of Revascularization: Where Are We and Where Are We Heading?
Source: J Cardiovasc Transl Res. 2015 May 19;8(4):211–20. doi: 10.1007/s12265-015-9632-6 (PMC4473080; doi:10.1007/s12265-015-9632-6)
Supplement: Supplementary file 3 — (DOCX 22 kb) [file 12265_2015_9632_MOESM3_ESM.docx]

Supplementary Table 3: Recommendations for antithrombotic treatment in ST-segment Elevated Myocardial Infarction patients undergoing PCI:

|  | **ESC GL 2014** | | **ESC GL 2010** | | **American Societies GL** | |
| --- | --- | --- | --- | --- | --- | --- |
| Antiplatelet Therapy | ASA is recommended for all patients without contraindications and continued long-term regardless of treatment strategy. | **I A** | ASA | **I C** | ASA in patients already taking daily aspirin and in patients not on aspirin | I B |
|  |  |  |  |  | ASA after PCI should be continued indefinitely | I A |
|  | P2Y_12_ Inhibitor is recommended in addition to ASA and maintained over 12 months unless contraindicated (eg, excessive bleeding risk), options are: | **I A** | n.a. | **-** | A loading dose of P2Y_12_ Inhibitor should be given to patients undergoing PCI with stenting, options include: | **I A** |
|  | Prasugrel, if no contraindications. | **I B** | Prasugrel | **I B** | Prasugrel | **I B** |
|  | Ticagrelor, if no contraindications. | **I B** | Ticagrelor | **I B** | Ticagrelor | **I B** |
|  | Clopidogrel, only when prasugrel or ticagrelor are not available or contraindicated. | **I B** | Clopidogrel (600 mg loading dose as soon as possible) | **I C** | Clopidogrel | **I B** |
|  | It is recommended to give P2Y_12_ inhibitors at the time of first medical contact | **I B** | n.a. | **-** | A loading dose of a P2Y12 receptor inhibitor should be given as early as possible or at time of primary PCI to patients with STEMI | **I B** |
|  | GP IIb/IIIa antagonist should be considered for bailout or evidence of no-reflow or a thrombotic complication. | **IIa C** | GP IIb/IIIa antagonists (in patients with evidence of high intracoronary thrombus burden): |  | GP IIb/IIIa inhibitor is reasonable at the time of PCI in patients treated with UFH whether or not pre-treated with clopidogrel. | **IIa C** |
|  |  |  | Abciximab | **IIa A** | Abciximab administered in patients undergoing primary PCI, may be reasonable to administer intracoronary | **IIb B** |
|  |  |  | Eptifibatide | **IIa B** |  |  |
|  |  |  | Tirofiban | **IIb B** |  |  |
|  | Upstream used of GPIIb/IIIa antagonists (vs. in-lab use) may be considered in high-risk patients undergoing transfer for primary PCI | **IIb B** | Upstream GP IIb/IIIa antagonists | **III B** | Routine pre-catheterization laboratory (eg, ambulance or emergency department) administration of GPIIb/IIIa inhibitors as part of an upstream strategy for patients with STEMI undergoing PCI is not beneficial | **III B** |
| Anticoagulant Therapy | Anticoagulant therapy is recommended for all patients in addition to antiplatelet therapy during PCI | **I A** | n.a. | **-** | An anticoagulant should be administered to patients undergoing PCI | **I C** |
|  | Anticoagulation is selected according to both ischemic and bleeding risks, and according to the safety-efficacy profile of the chosen agent | **I C** | n.a. | **-** | n.a. | **-** |
|  | Bivalirudin (0.75 mg/kg bolus + 1.75 mg/kg/h up to 4 hours after PCI) | **IIa A** | Bivalirudin (monotherapy) | **I B** | Bivalirudin is useful as an anticoagulant, for patients undergoing PCI with or without prior treatment with UFH | **I B** |
|  | UFH | **I C** | UFH | **I C** | UFH is useful in patients undergoing PCI | **I C** |
|  | Enoxaparin with or without GPIIb/IIIa inhibitor. | **IIa B** | n.a. | **-** | n.a. | **-** |

GL: Guidelines

This table is based on the original table present in the ESC revascularization guidelines 2014. The data regarding the 2010 guidelines and American guidelines are adapted to allow comparison. The fields in which the comparison was not possible or could risk to distort the sense of the indications have been specified as “not applicable” (n.a.)
